# Combining the benefits of 3D acquisitions and spiral readouts for VASO fMRI at UHF

**DOI:** 10.1162/imag_a_00308

**Published:** 2024-10-07

**Authors:** Alejandro Monreal-Madrigal, Denizhan Kurban, Laurentius Huber, Dimo Ivanov, Nicolas Boulant, Benedikt A. Poser

**Affiliations:** Maastricht Brain Imaging Centre, Faculty of Psychology and Neuroscience, Maastricht University, Maastricht, The Netherlands; National Institutes of Mental Health, Bethesda, MD, United States; University-Paris-Saclay, CEA, CNRS, BAOBAB, NeuroSpin, Gif-sur-Yvette, France

**Keywords:** non-BOLD fMRI, CBV fMRI, ultra high field, non-Cartesian readouts, pulseq, open-source

## Abstract

We present a slice-saturation slab-inversion VASO (SS-SI-VASO) sequence with a 3D stack-of-spirals readout implemented in Pulseq and show that it can accurately capture changes in cerebral blood volume. Its performance is compared to a state-of-the-art SS-SI-VASO sequence with a 3D EPI readout. We observed an increase in tSNR and improvement in z-scores in spiral compared to 3D EPI acquisition, demonstrating that spiral readouts are suitable for CBV-weighted laminar fMRI. Additionally, we found an increase in sensitivity and relative specificity with the proposed method using spiral readouts, compared to EPI readouts. Several correction approaches were employed in the spiral reconstruction to improve image quality. Incidentally, BOLD contrast in the proposed short-TE spirals is almost as high as that of the 3D EPI at longer TE. In this work, we demonstrate that spiral readouts are promising, especially in applications where there is a need for short TE, such as mesoscopic fMRI at higher fields. The vendor-agnostic Pulseq implementation of VASO, together with an open-source reconstruction framework, aims at increasing the availability and utilization of VASO in high-resolution fMRI experiments.

## 1 Introduction

Functional MRI (fMRI) allows to non-invasively measure brain activity by detecting neurally driven hemodynamic signal changes. One of its main advantages is thus the high spatial resolution (on the order of mm) compared to electrophysiological measures like EEG, at the expense of lower temporal resolution (on the order of seconds). Recent advances in hardware and acquisition techniques have made it possible to acquire fMRI data at sub-millimeter spatial and sub-second temporal resolutions and to study neuronal activation at the level of cortical layers and columns (Bollmann & Barth, 2021;Feinberg et al., 2018). fMRI uses the fact that neural activation triggers a response in the hemodynamic parameters cerebral blood flow (CBF), cerebral blood volume (CBV), and cerebral metabolic rate of oxygen (CMRO_2_). The most widely used fMRI technique is the blood oxygenation level-dependent (BOLD) contrast (Ogawa et al., 1990) that measures a combination of CBF, CBV, and CMRO_2_and is sensitive to the resulting net change in local deoxyhemoglobin concentration.

BOLD captures changes in the local magnetic field caused by the paramagnetic nature of deoxygenated hemoglobin and is therefore primarily sensitive to the draining vasculature downstream to the site of neuronal activation (Polimeni et al., 2010). Methods directly measuring CBF, CBV, and/or CMRO_2_can therefore be more spatially specific than BOLD, which makes them attractive auxiliary measures in high-resolution laminar and columnar fMRI. CBV and CBF are quantitative measures of brain activity, while BOLD is not (L. Huber et al., 2019).

Vascular Space Occupancy (VASO) measures CBV using blood itself as an endogenous contrast agent (Lu et al., 2013). The VASO contrast arises predominantly from the microvessels that expand due to local neural activation. An MR signal change between activation and rest is created in VASO fMRI by selectively nulling the blood signal during excitation by a preceding inversion pulse, taking advantage of a small T_1_difference between capillary blood and the surrounding tissue. In this way, the signal change originates primarily from the parenchyma (tissue + microvasculature) and a signal decrease is observed during activity when the blood volume is increased and hence a larger fraction of the voxel volume nulled. Changes in the MR signal during rest and activity can then be associated with changes in CBV. Over the past few years, VASO has become very attractive for high-resolution layer fMRI studies at ultra-high field (UHF) since it provides a favorable compromise between sensitivity and specificity (L. Huber et al., 2019).

The use of VASO fMRI at UHF still poses several challenges (L. Huber et al., 2018). Three of the most important ones are unwanted*BOLD contamination*, limited*detection sensitivity*, and poor temporal*sampling efficiency*.*BOLD contamination*can be removed from the VASO contrast by division with a BOLD-weighted volume acquired immediately after the VASO one (L. Huber et al., 2014), while 3D readouts increase*detection sensitivity*(L. Huber et al., 2018). Current VASO methods use Cartesian Echo-Planar Imaging (EPI) to increase*sampling efficiency*, allowing acquiring a whole k-space plane in one excitation. Nevertheless, given the low Signal-to-Noise Ratio (SNR) nature of the VASO contrast, a short Echo Time (TE) is desired. The shortest achievable TE with current EPI implementations for high-resolution data, including in-plane acceleration and Partial Fourier, is on the order of tens of milliseconds.

Alternatives to Cartesian readouts to enable shorter TE are non-Cartesian acquisitions such as radial and spiral trajectories (Delattre et al., 2010). With these sampling strategies, k-space points do not fall onto a Cartesian grid anymore. Spiral sampling has improved sampling efficiency since it covers more k-space per unit time compared to EPI. Its benefits are counterbalanced by some implementation challenges. Since the sampling points do not fall onto an equidistant grid, the use of the Fast Fourier Transform (FFT) algorithm (Cochran et al., 1967) for reconstruction is not possible, and the reconstruction of non-Cartesian data usually makes use of more computationally demanding algorithms such as the Non-Uniform Fast Fourier Transform (NUFFT) (Fessler & Sutton, 2003). Images reconstructed from non-Cartesian acquisitions exhibit different off-resonance artifacts (compared to EPI), such as blurring due to B_0_magnetic field inhomogeneities and ring artifacts; the point spread function is also adversely affected and higher order field dynamics are present (Delattre et al., 2010). Therefore, methods to correct for static field inhomogeneities and field dynamics need to be included in the reconstruction (Barmet et al., 2008).

The most common and simplest design for a spiral trajectory is an Archimedean spiral. Some spiral parameters can be adjusted to achieve different acquisition and reconstruction results; the most important parameters are variable density α, that defines how the center of the k-space is sampled more densely than the outer part (Delattre et al., 2010), in-plane under-sampling (R_xy_), number of in-plane shot readouts and plane rotations when using 3D stack-of-spirals (3D spiral). Designing an efficient spiral trajectory must consider the scanner hardware limitations (gradient amplitude, slew rate, and mechanical resonances) as well as subject safety limits such as Peripheral Nerve Stimulation (PNS) (Delattre et al., 2010). An efficient algorithm for spiral design that considers scanner hardware limitations has been proposed inLustig et al. (2008). Since functional imaging protocols aim to capture dynamic variations of the MRI signal, single-shot spiral-out readouts are attractive alternatives to EPI ones.

For VASO fMRI, the ideal sequence would have (i) a short TE to maximize SNR and reduce BOLD effects, and (ii) an efficient sampling readout to allow the acquisition of as many slices or partitions around the blood nulling point as possible. This makes spirals attractive because they fulfill both conditions. A recently proposed expanded signal model (Kasper et al., 2018), advances in image reconstruction techniques, and field monitoring have made possible the use of spirals for functional MRI. Some previous work at 7T has successfully used 2D spiral readouts for BOLD (Kasper et al., 2022) and ASL (Kurban et al., 2022) contrasts.

The goal of this work was to improve the sensitivity, sampling efficiency, and availability of VASO for laminar fMRI. We achieved this by combining the benefits of spiral readouts and 3D acquisitions using open-source software. We combined the slice-saturation slab-inversion VASO or SS-SI-VASO (L. Huber et al., 2014) contrast with a 3D single-shot (in-plane) spiral-out stack-of-spirals readout, to improve the laminar sensitivity and sampling efficiency. The use of open-source software aims at improving the availability of this method. We refer to this method as 3D spiral VASO fMRI.

## 2 Methods

### 2.1 Image reconstruction theory

Single-shot spiral-out acquisitions allow for a short TE (few milliseconds) and offer benefits for lower-SNR methods such as VASO especially when high spatial resolutions are desired. These sampling strategies typically require long readout times and suffer from off-resonance effects due to B_0_-field inhomogeneity, which are amplified at UHF. To take these imperfections into account, an off-resonance frequency term must be included in the SENSE signal model (Pruessmann et al., 1999)

$$s_{\gamma }\left(t\right)=\int_{V}c{\left(r\right)}_{\gamma }m\left(r\right)e^{-ik\left(t\right)r}e^{-i\Delta \omega_{0}\left(r\right)t}dV$$ (1)

wheres(t)is the k-space data from each receiver channel*ɣ*,c(r)is the complex spatial sensitivity of the*ɣ*coil,m(r)is the magnetization,k(t)is the k-space trajectory, and the term$\Delta \omega_{0}$denotes the angular off-resonance frequency which is proportional to field inhomogeneities. Several low-rank approximation methods for off-resonance correction have been proposed, such as time-segmented (Noll et al., 1991), multi-frequency interpolation (Man et al., 1997), and gridding approaches (Knopp et al., 2009).

Discretizingequation 1and constructing a linear system of equations from the k-space data, we arrive at the matrix representation

$$s=Em,E=c{\left(r\right)}_{\gamma }e^{-ik\left(t\right)r}e^{-i\Delta \omega_{0}\left(r\right)t}$$ (2)

This inverse problem can be solved iteratively with the regularized least-squares cost function

$$\hat{m}=argmin\frac{1}{2}{\left\|Em-s\right\|}_{2}^{2}+\beta R\left(m\right)$$ (3)

where β is the regularization parameter andR(m)is a regularization function that is used to ensure that the algorithm converges to a stable solution. Several regularization approaches have been proposed and are still an important research area in the MRI community. Different algorithms exist to solveequation 3, and some of the most widely used are CG-SENSE (Pruessmann et al., 2001), ADMM (Boyd et al., 2011), and FISTA (Beck & Teboulle, 2009).

### 2.2 Data acquisition

Five healthy volunteers were scanned on a ‘classic’ Siemens Magnetom 7T whole-body scanner (Siemens Healthineers, Erlangen, Germany), equipped with a 1Tx-32Rx head coil (Nova Medical, Wilmington, MA, USA) and an SC72 gradient system with 70 mT/m peak amplitude and 200 T/m/s slew rate. All volunteers provided written informed consent following the protocols of the local ethics committee. Each volunteer underwent two fMRI runs performing a visuo-motor task acquired using the 3D spiral and 3D EPI readouts separately in one session. Each run was approximately 10 minutes.

An SS-SI-VASO sequence (L. Huber et al., 2014) with a 3D spiral readout was implemented using Pulseq (Layton et al., 2017). The VASO inversion pulse consisted of a 10 ms time resampled frequency offset corrected inversion (TR-FOCI) pulse (Hurley et al., 2010), applied 600 ms before the start of the VASO readout module; this way, the center partition of the k-space was measured around the blood nulling point. For BOLD contamination correction, a BOLD-weighted volume was acquired right after the VASO one. A fat saturation module was used before each partition readout to suppress the fat signal. The single-shot spiral-out readout was designed to achieve the desired k-space trajectory in the shortest time within gradient constraints, using the optimal gradient design algorithm (Lustig et al., 2008). The spiral was designed with G_max_= 35 mT/m, SR_max_= 155 mT/m/ms and BW = 500 MHz. The spiral readouts were stacked in the k_z_direction to obtain the 3D spiral readout.

For comparison, a state-of-the-art 3D EPI sequence for VASO fMRI (Stirnberg & Stöcker, 2021) was used. Similar to the spiral approach, it consists of an SS-SI-VASO implementation with a TR-FOCI inversion pulse, a fat saturation module per partition, and a BOLD-weighted volume acquired after the VASO one for BOLD correction. A schematic depiction of the sequence diagrams of the 3D spiral and 3D EPI can be found inFigure 1.

**Fig. 1.**
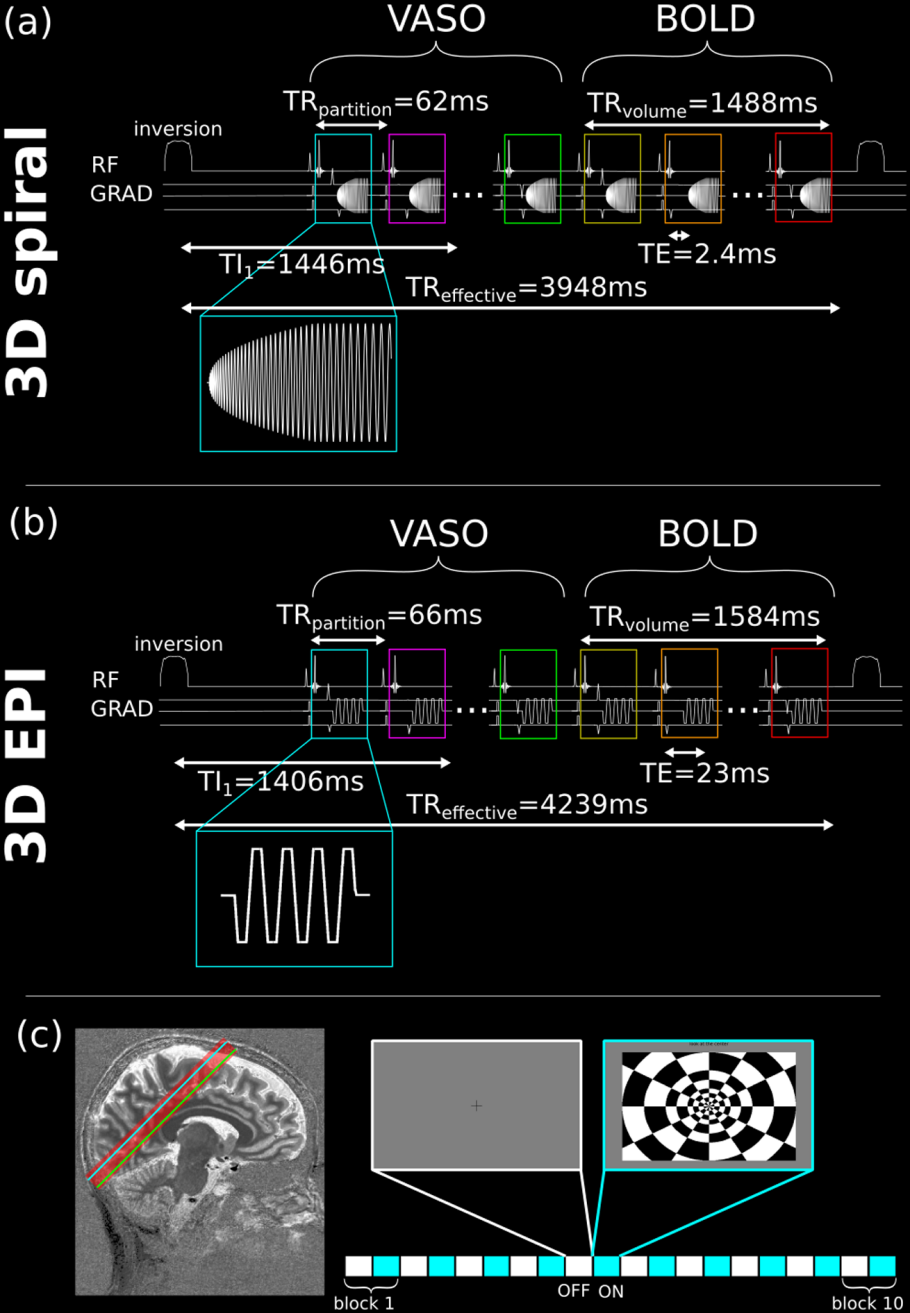
Sequence diagrams and paradigm design. (a) Schematic depiction of sequence diagram of the 3D stack-of-spirals acquisition (3D spiral). A TR-FOCI inversion pulse is applied 600 ms before the start of the excitation of the VASO volume. A BOLD-weighted volume is acquired immediately afterwards, to be used for BOLD correction. A fat saturation module is used before each partition readout; (b) sequence timing diagram of the 3D EPI acquisition; and (c) FOV, orientation, and paradigm block-design (blue and green lines represent the locations of partitions shown in later figures).

The parameters of the spiral acquisition were: FOV = 192 x192 x 24 mm^3^, res_nominal_= 0.8 x 0.8 x 1.0 mm^3^, 24 k_z_partitions with linear encoding order, TE = 2.4 ms, TI_1_= 1446 ms, TR_partition_= 62 ms, TR_volume_= 1488 ms, spiral variable density α = 1.6, and in-plane under-sampling r_xy_= 3, resulting in a spiral readout duration of 52 ms. The parameters of the 3D EPI acquisition were: FOV = 192 x 192 x 24 mm^3^, res_nominal = _0.8 x 0.8 x 1.0 mm^3^, 24 k_z_partitions with linear encoding order, TE = 23.5 ms, TI_1_= 1406 ms, TR_partition_= 66 ms, TR_volume = _1584 ms, 1.02 ms echo spacing, BW = 1096 Hz/px, GRAPPA 3, and 6/8 partial Fourier, resulting in a 63 ms readout duration. Both 3D EPI and 3D spiral used a sinc RF pulse of 2.56 ms for excitation, set at the Ernst Angle. The resulting effective temporal resolutions for the spiral and EPI sequences were TR_effective_= 3948 ms and TR_effective_= 4239 ms, respectively.

To calculate the sensitivity and B_0_off-resonance maps, 2D low-resolution multi-echo gradient echo (ME-GRE) scans with matched coverage and slice positions as the functional runs were acquired before each spiral run. The following acquisition parameters were used: res = 1.6 x 1.6 x 1 mm^3^, TE_n_= 2.72, 6.31, 12, 25, and 32 ms, nominal flip angle = 25°, and TR = 1000 ms. For B_0_estimation, only the first 3 echoes were used since they provided the best fieldmap estimate and image quality.

### 2.3 Paradigm design

The stimulus consisted of a full-screen flickering (approx. 8 Hz) black and white radial checkerboard consisting of 30 s OFF (rest) and 30 s ON (active). The duration of each functional run was set to 10 minutes for both spiral and Cartesian acquisitions. Given the different effective temporal resolution of spiral and Cartesian EPI, the block design consisted of 8 TRs of rest followed by 8 TRs of activation for spiral acquisitions, and 7 TRs of rest followed by 7 TRs of activation for Cartesian EPI. Each functional run consisted of 160 and 140 volumes for spiral and Cartesian EPI, respectively, for a total of 10 blocks. During activity periods, participants performed a thumb-to-digits tapping with both hands while the visual stimulus was presented. During rest periods, participants fixated on a cross at the center of the screen.

### 2.4 Spiral image reconstruction

To account for 0^th^order field dynamics resulting from the gradients playing the spiral readout, the Eddy Current Compensation phase applied by the scanner (which differs from the measured one) was removed from the spiral raw data, the phase terms being obtained from simulations using the nominal gradient waveforms. After this, the raw data were demodulated with the zero order k_0_measurements obtained with a Skope (Skope MRT, Zürich, Switzerland) clip-on field camera (Barmet et al., 2008) from a separate scan. To account for scanner drift during the functional run, partial Dynamic Off-resonance Correction in K-space (DORK) (Pfeuffer et al., 2002) was also applied to the raw data. Here, the first samples (before the readout gradient is played) of the center partition (without phase encoding gradient) were used as Free Induction Decay (FID) navigators, the phase of these navigators was compared to a reference one (from the start of the scan), and any phase difference was removed from the raw data of each volume. The corrected spiral raw data were subsequently merged with the nominal trajectory obtained from Pulseq into an MRD file (Inati et al., 2017). The choice to use the nominal trajectory for reconstructions was driven by the small error and no significant image quality difference between measured and nominal trajectory as shown inSupplementary Figure 1.

The low-resolution ME-GRE scan was used to obtain the sensitivity maps$c{\left(r\right)}_{\gamma }$and B_0_off-resonance maps using the ESPIRiT (Uecker et al., 2014) implementation of MRICoilSensitivities.jl and regularized field map estimation (Lin & Fessler, 2020) algorithm as implemented in MRIFieldMaps.jl.

For spiral image reconstruction, the MRIReco.jl (Knopp & Grosser, 2021) package was used with an ADMM solver, “L1” regularization, and density compensation function (Pipe & Menon, 1999). Off-resonance correction followed the time-segmented gridding approach described inEggers et al. (2007). This method exploits the similarity between methods used for non-Cartesian sampling such as regridding and off-resonance correction ones, and both of them solve the problem of approximating an exponential function. With this method, the k-space samples and the image pixels are embedded into a higher dimensional space followed by reconstruction with Non-Uniform FFTs. The number of time segments used in this work was 24. The MRD file, sensitivity, and B_0_maps together with sequence parameters obtained from Pulseq were the input for the iterative CG-SENSE reconstruction in MRIReco.jl. The 3D EPI data were reconstructed using the vendor software from the scanner. A complete overview of the 3D spiral pipeline can be found inFigure 2.

**Fig. 2.**
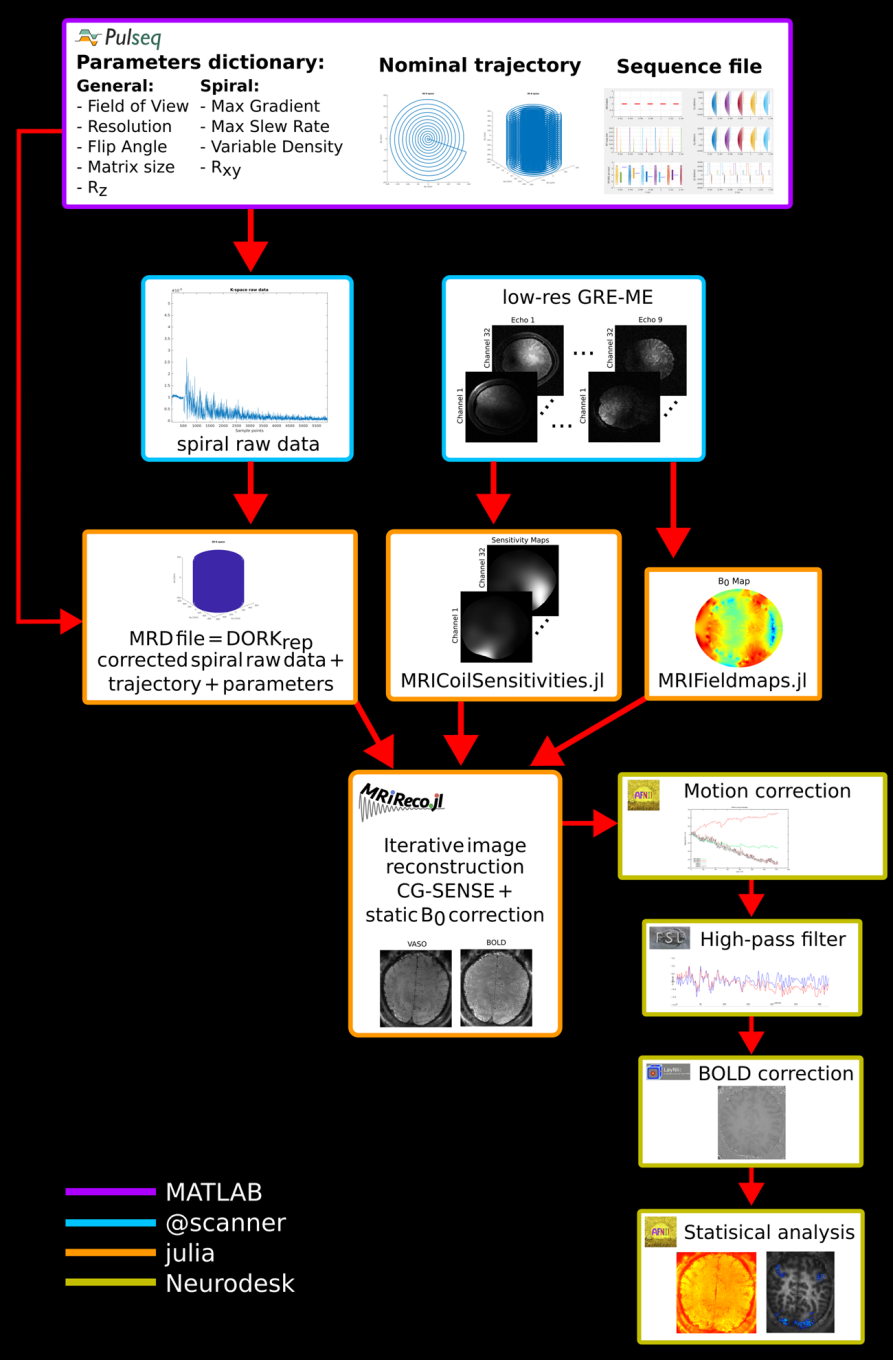
Overview of the 3D spiral SS-SI-VASO acquisition, reconstruction, and analysis pipeline. Acquisition**:**The Pulseq sequence is generated in MATLAB; nominal trajectory and parameters dictionary are saved. Spiral and low-resolution GRE data with matched parameters are acquired. Reconstruction**:**The DORK-corrected spiral raw data are merged with the nominal trajectory and other parameters into an MRD file. Sensitivity and B_0_maps are calculated from the low-resolution GRE scan. The MRD file, sensitivity, and B_0_maps are the input to the reconstruction in MRIReco.jl. Analysis**:**Rigid motion correction is performed with AFNI, high-pass filter in FSL, and BOLD correction in LayNii, and the statistical analysis is done with AFNI, all this using Neurodesk.

Reconstruction was performed on a dedicated server running Ubuntu 18.04, with 1 TB of memory and two Intel Xenon Scalable Processor “Skylake” Gold 6140 with 18 cores each, for a total of 36 cores.

### 2.5 Data analysis

The same data analysis pipeline implemented in Neurodesk (Renton et al., 2024) was used for both 3D spiral and 3D EPI datasets (Fig. 2):*Motion correction*was performed using AFNI. The time series was registered to the first volume of the acquisition. The same parameters were used to correct for the VASO and BOLD time-series separately. A*high-pass temporal filter*was applied to the data using FSL with an FWHM of the duration of rest/activity blocks.*BOLD correction*was achieved by dynamic division of the images with and without blood nulling and it was performed with the function LN_BOCO from LayNii (L. (Renzo)Huber et al., 2021) after temporal up-sampling of both VASO and BOLD time-series. Temporal up-sampling is needed to estimate the expected BOLD contamination during the acquisition of the VASO volumes, efficiently doubling the number of volumes. Mean time-series and tSNR were computed as quality metrics for both the VASO and BOLD images using AFNI and LayNii. Activation maps were computed using a General Linear Model (GLM) and clustered using AFNI 3dclust. No spatial Gaussian smoothing was applied.*Activation maps*were obtained for the VASO and BOLD time-series separately.*Layers*were estimated with the LayNii command LN_GROW_LAYERS and a manually created rim covering an ROI on V1 after spatial up-sampling; layer profiles were calculated as the mean of z-score values in each layer using AFNI.

The SS-SI-VASO sequence shares some similarities with MP2RAGE; two volumes are acquired with different inversion times. Since both images share the same readout, the T_2_* (BOLD) weighting, the proton density weighting, and the bias fields are expected to be identical. Solely, the T_1_-weighting is different. Analogously to MP2RAGE processing, in VASO a T_1_-weighted volume can be obtained by computing the inverse coefficient of variation of the VASO and BOLD volumes concatenated using the 3dTstat -cvarinv command from AFNI (Cox, 1996) – structural contrast that is dominated from signal changes between the respective inversion times. This T_1_-weighted volume is commonly estimated in all layer-fMRI VASO pipelines of the literature, it is inherently aligned to the functional data, and it can be used as underlay for activation maps without the need of a separate T_1_-weighted reference scan.

## 3 Results

Reconstructions of 3D spiral and 3D EPI images of the functional run, as well as the GRE reference image for subject 1 are shown inFigure 3. It can be seen that spiral images closely follow the geometry of the low-resolution GRE acquisition, demonstrating that the proposed implementation, correction strategies, and reconstruction generate accurate MR images.

**Fig. 3.**
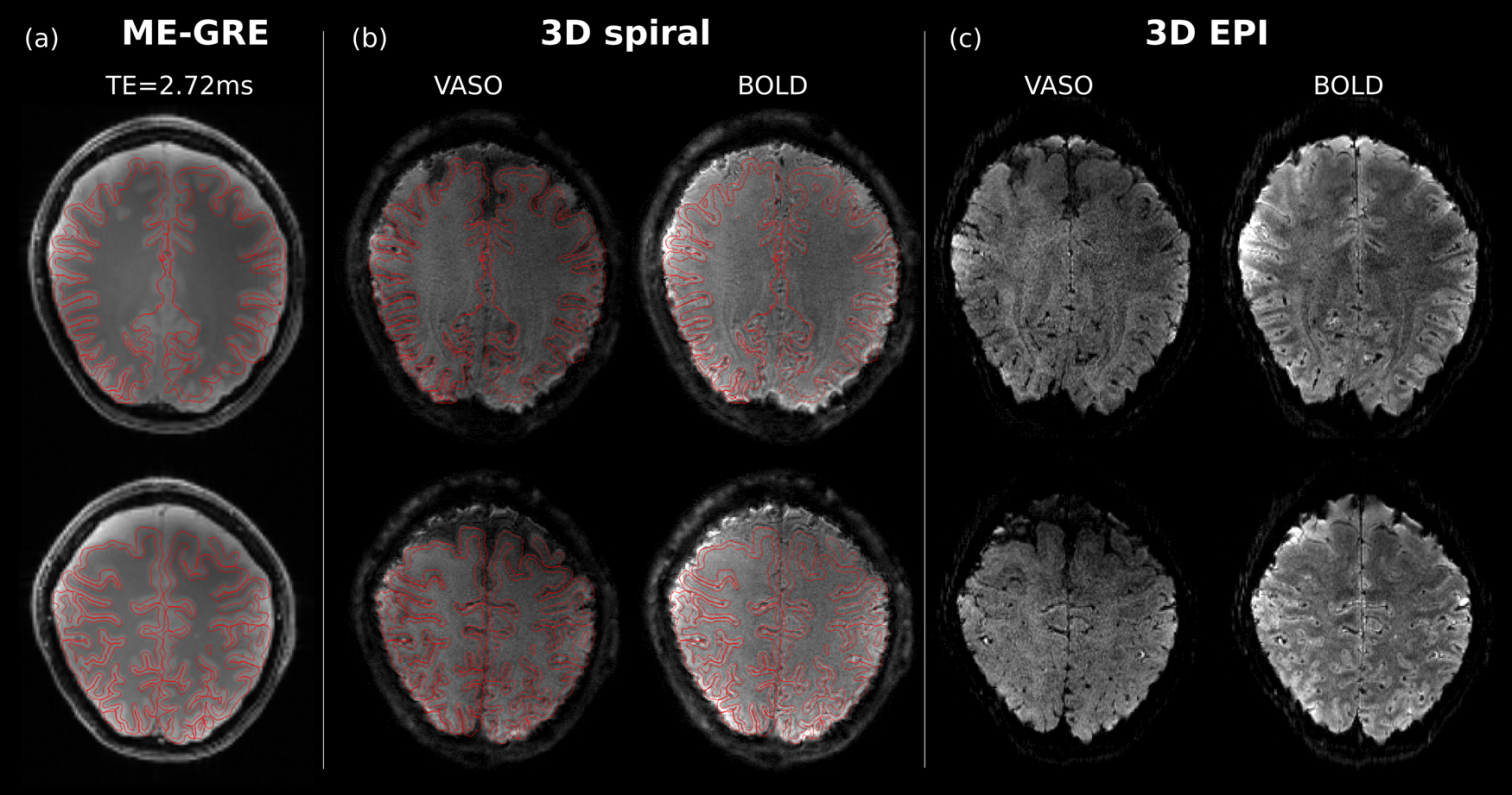
Quality of functional run images. (a) First echo (TE = 2.72 ms) of the low-resolution ME-GRE used for sensitivity and B_0_map calculation, (b) and (c) fully-corrected single volume of VASO and BOLD from the functional runs of the 3D spiral and 3D EPI, respectively. The overlay red lines of the spiral images show that the proposed sequence properly captures the imaging volume without significant geometric distortions. The same lines also exhibit that some areas especially at the air-tissue interface present artifacts due to incomplete off-resonance correction.

**Fig. 4.**
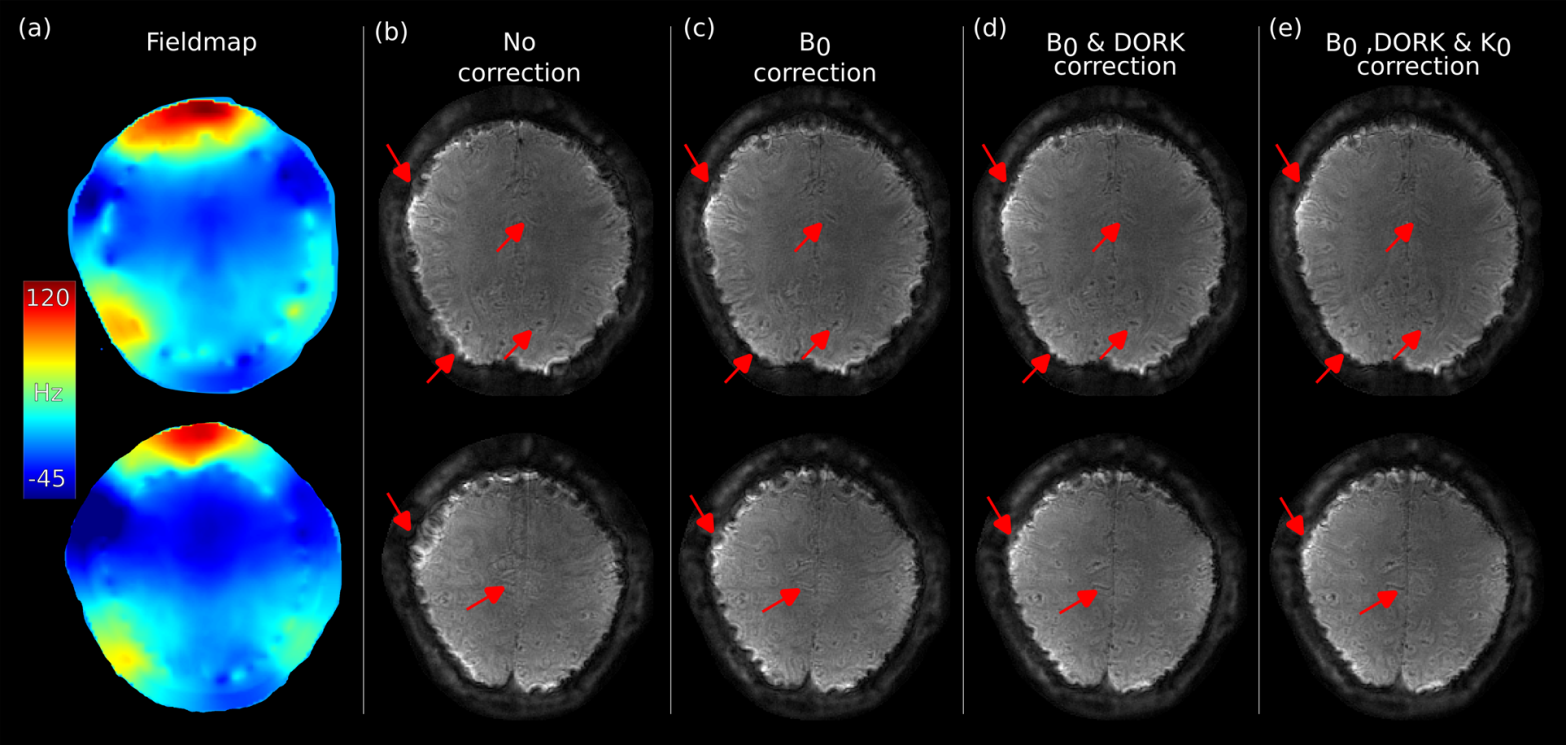
Corrections of spiral data. (a) B_0_map of two different slices. A single BOLD volume after 9 minutes of the functional run, (b) the images without correction present severe artifacts (red arrows), (c) artifacts are reduced after performing B_0_correction, (d) image quality is significantly improved when B_0_and repetition DORK correction are applied, and (e) a further improvement is achieved when performing k_0_correction.

As previously mentioned, spiral acquisitions with long readout duration are prone to artifacts due to static and dynamic field fluctuations.Figure 4shows the effect of the B_0_off-resonance correction of the spiral functional run after 9 minutes of scanning, it can be seen that severe off-resonance artifacts are present, and the majority of them can be removed with the off-resonance correction strategy used in this work. The same figure also shows the importance of performing DORK_rep_correction to correct for 0^th^order (scanner drift).

To assess the temporal stability of the functional run, mean timeseries, tSNR and tSNR/effective resolution (effective resolution was obtained from PSF simulations) maps of both Cartesian and spiral acquisitions were computed, and results for subject 1 can be found inFigure 5. A factor of 1.8 higher whole volume average tSNR for VASO and BOLD is achieved with the proposed single-shot spiral-out readout; this is expected given the short TE and reduced T_2_* signal decay. When taking effective resolution into account, the tSNR gain is a factor of 1.4 higher for 3D spiral. Comparable improvements in tSNR values are observed for all subjects.

**Fig. 5.**
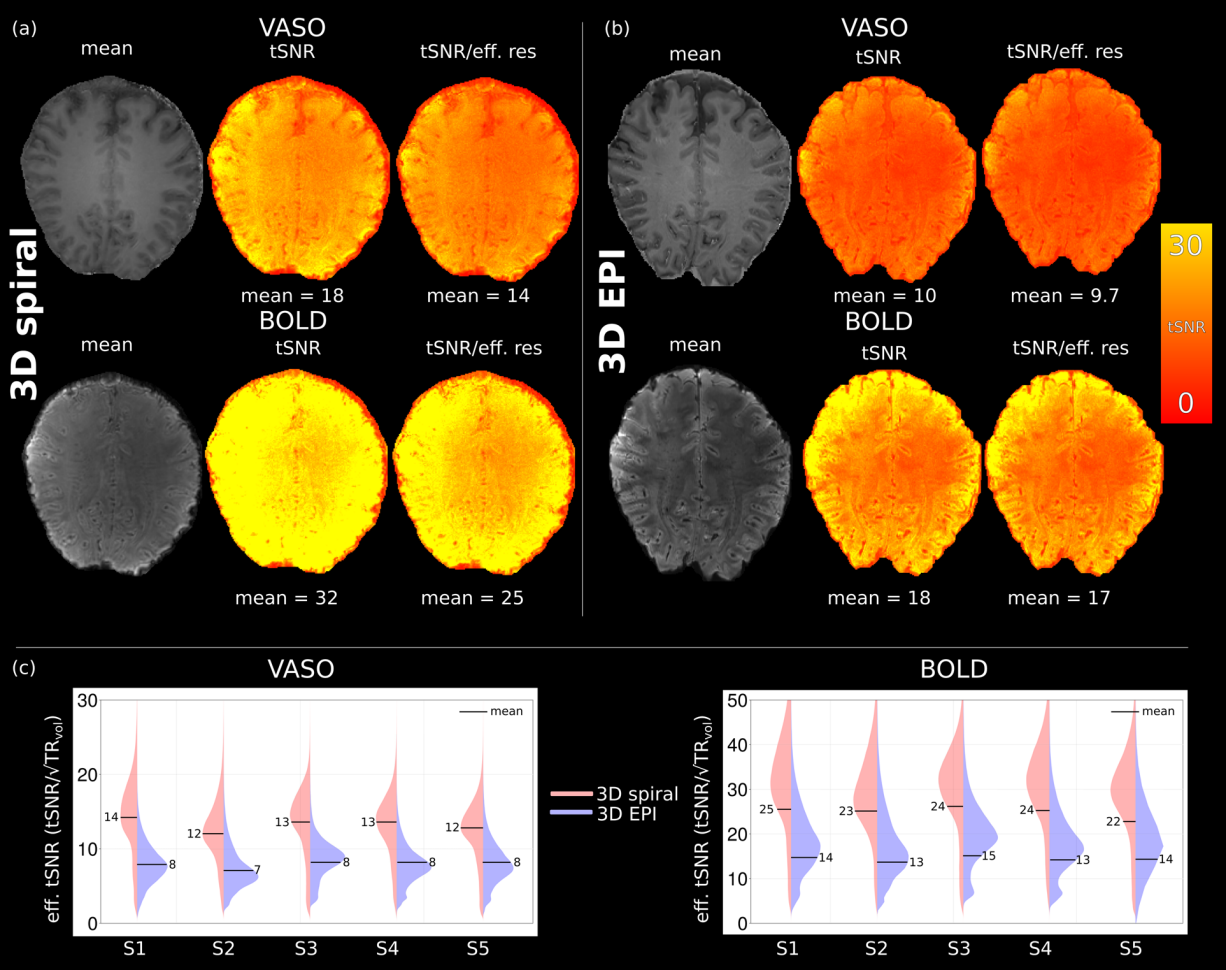
Masked VASO and BOLD mean time series and tSNR maps (subject 1) from (a) spiral and (b) Cartesian acquisitions. A factor of 1.8 higher whole volume average tSNR for spiral VASO and BOLD is achieved with the proposed implementation, and this is reduced to 1.4 when taking effective resolution into account. (c) Per subject analysis of effective tSNR, the ~1.8 factor gain in tSNR is achieved in all subjects.

A comparison between activation maps from 3D EPI and 3D spiral acquisitions for both BOLD and VASO of subject 1 is shown inFigure 6, and it can be seen that the activation pattern obtained with the proposed implementation closely follows the one obtained with the Cartesian state-of-the-art sequence. It should also be noted that there are fewer active voxels of the BOLD contrast in the spiral case compared to the EPI, and this is expected to be an effect of the smaller BOLD contribution given the short TE of the spiral approach. The time-series plots show that both the Cartesian and the proposed spiral implementation capture signal changes during the block-design while activation patterns closely follow the rest and activity periods.

**Fig. 6.**
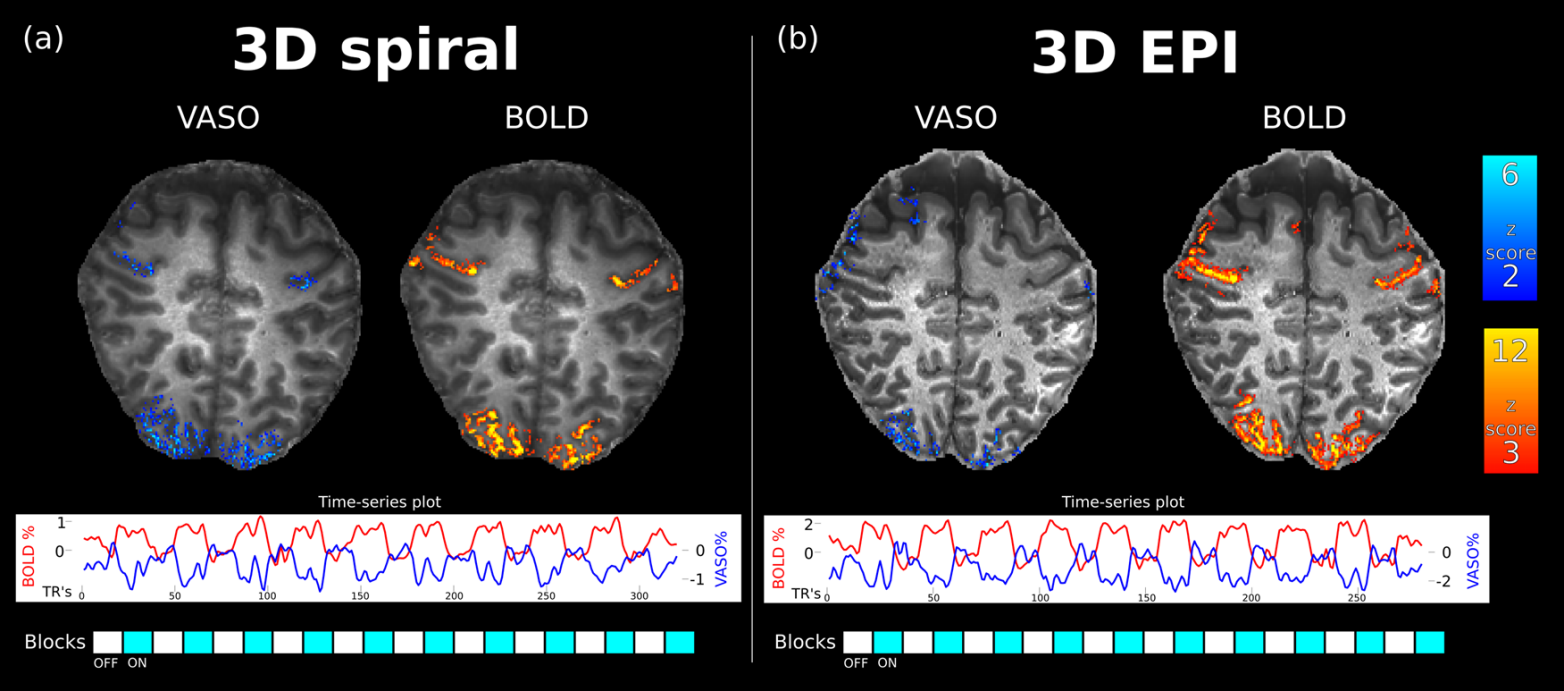
Clustered activation maps obtained with (a) 3D spiral and (b) 3D EPI for the VASO fMRI experiment. Timecourse plot shows the normalized signal timecourse averages over visual and motor active voxels. Note that VASO (blue line) is a negative CBV contrast with an expected signal decrease during activity.

To validate that our implementation accurately captures changes in CBV and that the benefits of the VASO contrast are observed, layer profiles of an ROI in the visual cortex for the 3D spiral and 3D EPI data were obtained. Results of subject 1 can be found inFigure 7a, and it can be seen that in both cases the BOLD activity is more weighted towards the CSF and the VASO activity is not as expected. The figure also shows an overview of the percentage of active voxels (after clustering) out of the total voxels of the brain area. It can be observed that the percentage of active voxels is greater for the VASO contrast of the 3D spiral compared to the 3D EPI. For the BOLD contrast, the opposite holds, more activity is observed in the 3D EPI approach than the 3D spiral one. This shows the higher detection sensitivity of the proposed approach in comparison to the state-of-the-art 3D EPI for VASO fMRI. The boxplots inFigure 7show that the z-scores are higher in all but one subject for the 3D EPI case, and this suggests that the CNR is higher than for the 3D spiral.

**Fig. 7.**
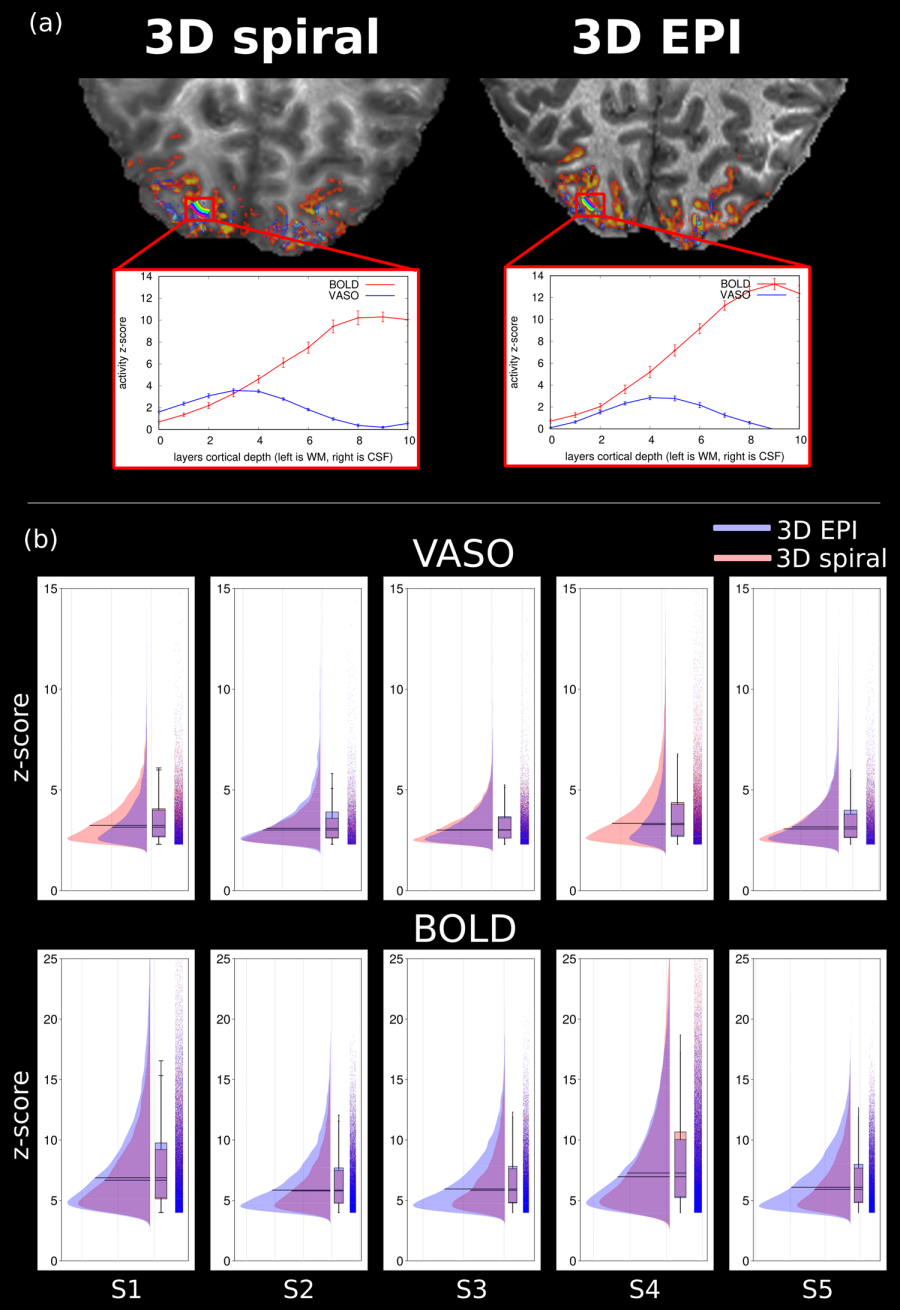
Layer profiles and individual subject analysis of z-scores. (a) Layer profiles (ROI in V1) of VASO and BOLD contrast for both 3D spiral and 3D EPI of subject 1. VASO is less weighted towards CSF in contrast to BOLD. (b) Raincloud plots showing VASO and BOLD distribution of active voxels after clustering and thresholding for both 3D EPI (blue) and 3D spiral (red). The histogram shows the percentage of active voxels out of the total matrix size for both spiral and EPI readouts relative to each other. For BOLD contrast, less voxels are active with the 3D spiral as compared to 3D EPI; the opposite holds for VASO where the 3D spiral approach has a higher number of active voxels than 3D EPI for all but one subject. Both effects are expected to be a consequence of the short TE of the 3D spiral method.

**Fig. 8.**
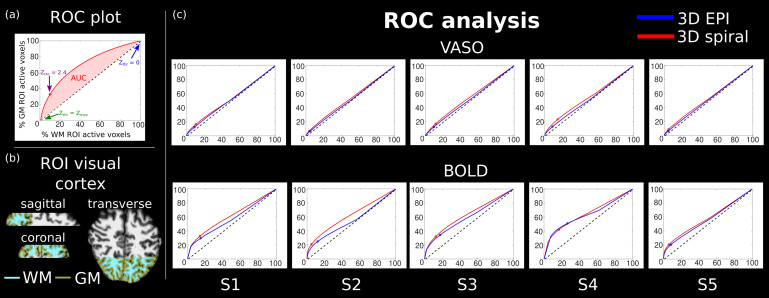
Measure of relative specificity using Receiver-Operator Characteristic (ROC) Curves. (a) Diagram explaining an ROC plot, (b) ROI in visual cortex used to compute ROC curves, and (c) ROC curves for all subjects; it can be seen that the area under the curve is slightly larger for 3D spiral compared to 3D EPI, and these results highlight the higher specificity of the proposed VASO sequence with 3D spiral readouts. Axes are the same as in (a).

As a measure of spatial specificity, we used the Receiver-Operator Characteristic Curves (ROC) as detailed inGraedel et al. (2021)to quantify the relative specificity of both sequences used in this work. We display the ratio of active voxels (after clustering) in GM (true positives) and active voxels in WM (false positives) over a range of z-score thresholds.Figure 8shows the results for all subjects, where it can be seen that the area under the curve for both VASO and BOLD contrasts is slightly larger for the 3D spiral approach compared to the 3D EPI; this suggests that the proposed implementation provides a higher relative specificity. The same figure also shows that for a threshold of 2.4, the 3D EPI approach outperforms the 3D spiral for BOLD contrast and the opposite hold for VASO; this is in line with the results presented inFigure 7.

## 4 Discussion

In this work, we presented an SS-SI-VASO sequence with a stack-of-spirals (3D spiral) readout implemented using Pulseq. We determined the stability of this sequence with in-vivo measurements and a block-design visuo-motor task. We showed that it can accurately capture changes in CBV and compared its performance with a state-of-the-art SS-SI-VASO sequence with a 3D EPI readout. We have shown that spiral readouts are suitable for laminar fMRI; the advantages of the VASO contrast were observed, with layer profiles showing higher activation within gray matter rather than towards CSF, whereas the BOLD contrast is more weighted towards large draining veins and CSF.

The spiral approach provides a higher tSNR compared to 3D EPI. While this is expected from the shorter echo time with reduced T_2_*decay, we consider that the higher tSNR could partially also be an effect of the point spread function (PSF) of spiral readouts and residual B_0_blurring after correction, although results from the ROC analysis suggest that the broader 3D spiral PSF does not adversely affect the spiral activation maps. We observed a small improvement in active voxels in all but one subject for the VASO contrast with the spiral compared to EPI. As expected from the much shorter spiral TE, the opposite holds for the BOLD contrast where fewer voxels were found active in the spiral data where nevertheless a considerable amount of activation was seen. Another advantage of the spiral readout is the shorter TR_effective_, in our particular protocol 291 ms (7 %) shorter than EPI, allowing for more volumes to be collected in the same scanning time. This could be further improved with the use of higher undersampling factors in the spirals, which were kept moderate in the current experiments. For all analyses performed, the first two slices of both 3D spiral and 3D EPI were discarded since they exhibit significant wrap around artifacts.

When designing a spiral readout, care must be taken to avoid spending much energy in the acoustic resonance bands of the gradient system. These bands are gradient specific and must be avoided as much as possible to prevent system damage. A typical echo-planar readout train consists mainly of one characteristic frequency peak, corresponding to 1/2ES. In contrast, a spiral readout consists of various peaks with one significantly higher in amplitude and the entire spectrum potentially overlapping with mechanical resonances of the gradient coil. To our knowledge, there is no clear guideline on how much energy can be dissipated in a given resonance band, but rapid transitions through different frequencies as in spirals can be considered less harmful than EPI trains depositing most of their energy in a forbidden band. In this work, we selected the spiral gradient amplitude and slew rate, making sure the main frequency peak lies outside of the acoustic resonance band of our gradient system (SC72; resonance frequencies = 550 and 1100 Hz with safe bands of 100 and 300 Hz respectively). The frequency spectrum and energy inside acoustic bands of the spiral and EPI gradient waveforms used in this work are presented inSupplementary Figure 2.

The single-shot spiral-out readout used in this study consisted of a readout time of 52 ms and proved to be prone to artifacts due to B_0_field inhomogeneities. Spirals are especially sensitive to these imperfections since two gradients are played during the readout. This leads to a phase accumulation in two directions that translates into blurring in the image domain. On the other hand, Cartesian imaging only varies one gradient during readout, so the phase accumulates in one direction causing a shift of the object. For this reason, B_0_off-resonance correction was applied to the spiral data. We also found that an additional k_0_demodulation of the raw k-space data with measurements from a field camera improved the image quality. Correcting for B_0_inhomogeneity and k_0_demodulation were not enough to obtain acceptable image quality in functional experiments. DORK correction of the functional run was also necessary to account for scanner drift. The current reconstruction pipeline does not account for concomitant gradients (Maxwell terms). These effects are small for acquisition near the gradient isocenter and especially at high field; however, for completeness, they should have been considered in the reconstruction.

We hypothesize that the remaining B_0_artifacts in the spiral images as shown inSupplementary Figure 3are from two different sources and related to each other: (i) sub-optimal shimming especially in the posterior area of the brain and (ii) ring artifacts similar to the ones reported inKasper et al. (2022)and described inBlock and Frahm (2005). The ring artifacts are expected to be a consequence of through-plane dephasing or incomplete B_0_mapping and off-resonance correction. To reduce the amount of artifacts in the 3D spiral images, better methods for B_0_correction are needed and will be further explored. In this work, we did not see any particular B_1_^ + ^field inhomogeneity effects since we make use of a low flip and a small field of view placed in an area where we do not expect to have significant B_1_^ + ^field inhomogeneity. If full brain coverage was desired, parallel transmission (pTx) would like be necessary to maintain the same level of robustness. One limitation of Pulseq is that currently it does not support pTx. Once this is available, it will be implemented into our sequence. Further image quality improvements can be expected when using navigator data for motion (Wallace et al., 2019) and dynamic distortion correction (Wallace et al., 2021) or including information from concurrent field monitoring to characterize dynamic field fluctuations in the reconstruction.

It is also noted that the spiral nominal resolution used in this work was 0.8 x 0.8 x 1.0 mm^3^, but given the broadening of the point spread function when using spiral sampling, the in-plane effective resolution is expected to be lower. According to previous analysis inFeizollah and Tardif (2023), the spiral in-plane effective resolution is expected to be ~1.5 x 1.5 mm^2^(WM) and ~1.3 x 1.3 mm^2^(GM) compared to ~1.35 x 1.35 mm^2^(WM) and ~1.2 x 1.2 mm^2^(GM) for EPI acquisition with 6/8 Partial Fourier. With this, the blurring in the 3D spiral data is expected to decrease the effective resolution by 11% (WM) and 8% (GM) compared to the 3D EPI. We performed PSF simulations for the 3D EPI and 3D spiral readouts using a single point in the center of the image and simulated the raw data using an inverse NFFT including a T_2_* decay of 25 ms using MRIReco.jl. Our results are in-line with the ones mentioned where we see a ~6% reduction in effective resolution with the 3D spiral approach compared to the 3D EPI. These results can be found inSupplementary Figure 4.

A limitation of the proposed approach is the reconstruction time of the 3D spiral data which is considerably longer than the Cartesian one and has to be performed off-line, the average time needed to reconstruct one spiral volume being 2 minutes and using ~100 GB of memory. Also, the current reconstruction framework would not allow to reconstruct the volumes on the Siemens reconstruction server, given the memory requirements.

Further improvement to the 3D spiral readout with higher undersampling factors towards achieving higher resolutions and/or volume coverage can be expected from incorporation of CAIPIRINHA-like offsets between the spiral planes, as well as protocol-dependent optimization of the spiral parameters. Segmented spirals can also be beneficial for this type of acquisitions; its shorter readout makes them less sensitive to B_0_inhomogeneties. With this, an improvement in image quality and signal fluctuations in the fMRI series is expected, though at the cost of a longer TR. Subspace reconstruction has recently been explored for various applications of spiral and other non-Cartesian acquisitions (Cao et al., 2022;Zhao et al., 2018); this type of reconstruction can also be beneficial for fMRI, but this is to be explored in future studies.

Advances on hardware can also be beneficial for spiral acquisitions; high-performance gradients (Feinberg et al., 2023) would allow faster readouts for less T_2_* decay, yet at a cost of more bandwidth and thus also penalty in acquisition SNR. A benefit might thus rather be expected from using higher segmentation factors (to reduce impact of off-resonance or T_2_* effects), rather than merely faster acquisitions. The use of concurrent off-resonance measurements for dynamic correction of field fluctuations and trajectory imperfections to higher order should also lead to further improvements of image quality and temporal stability.

A relevant finding of this study is that the BOLD contrast in the proposed spiral approach at TE = 2.4 ms is almost as high as the one of the 3D at TE = 23 ms. One of the reasons why we decided to explore a spiral-out readout for VASO was to avoid BOLD contamination. Our results suggest that the BOLD contribution to the signal is high even at short TE; this means that the BOLD contribution at the high spatial frequencies of the k-space has a significant impact on activation. This could open the possibility to perform high-resolution spiral-out BOLD experiments with a TE that differs from the commonly used TE = T_2_*. This observation has been recently explored inEngel et al. (2022), where the concept of BOLD PSF to characterize the BOLD sensitivity in TE-asymmetric trajectories such as spirals was introduced.

We anticipate that the spiral-out VASO could play an important role for some applications, such as event-related paradigms, imaging areas with strong intra-voxel dephasing such as the frontal or entorhinal cortex, resting-state experiments where the more homogeneous g-factor distribution of spiral imaging (Lee et al., 2021) can be beneficial, and especially for mesoscopic functional experiments at higher fields (>7T), since T_2_* decreases with higher field strengths and short TEs are required.

This vendor-agnostic implementation of VASO, together with the open-source reconstruction framework, aims at increasing the availability and use of VASO on high-resolution fMRI experiments. We envision that this framework could be used and improved by a larger community of fMRI users, no matter what scanner vendor and software version is available to them.

## 5 Conclusion

In this work, we investigated a 3D spiral implementation for VASO fMRI. We have shown that an implementation of a VASO fMRI with a 3D stack-of-spirals readout sequence can be achieved using open-source and freely available programs such as Pulseq, MRIReco.jl, and Neurodesk. An improvement on VASO activation voxel count and specificity can be expected with the 3D spiral approach. Here, we demonstrate that spiral readouts are promising, especially in applications where there is a need for short TE, such as mesoscopic functional experiments at higher fields such as 9.4T and 11.7T where T_2_* is shorter and traditional 3D EPI readouts become more challenging.

## Supplementary Material

Supplementary Material

## Data Availability

The source code, example sequences, and instruction files are available on github (https://github.com/monreal93/3D_SOSP_fMRI). We welcome feedback and requests for assistance regarding the code.
