# Supplementary material for: Combining the benefits of 3D acquisitions and spiral readouts for VASO fMRI at UHF
Source: Imaging Neurosci (Camb). 2024 Oct 7;2:imag-2-00308. doi: 10.1162/imag_a_00308 (PMC12290527; doi:10.1162/imag_a_00308)
Supplement: Supplementary Material [file imag_a_00308-supp.pdf]

Supplementary figures

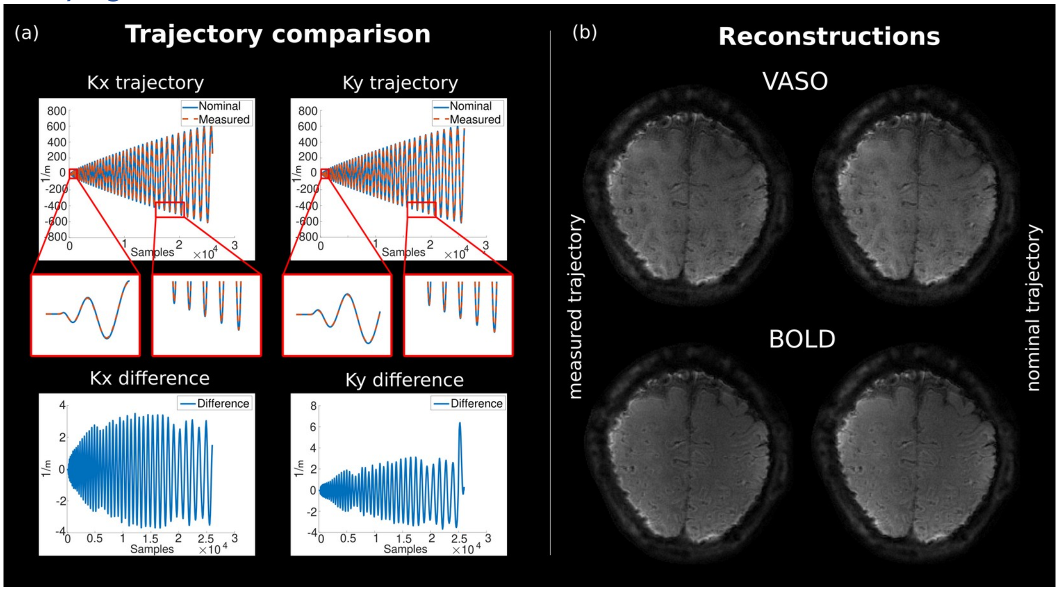

Supplementary figure 1: *Nominal and measured k-space trajectories comparison. (a) difference of k-space trajectories, the difference between nominal and measured is ~4 (1/m) and (b) reconstruction quality from both trajectories, no significant difference can be seen between reconstruction with both trajectories.*

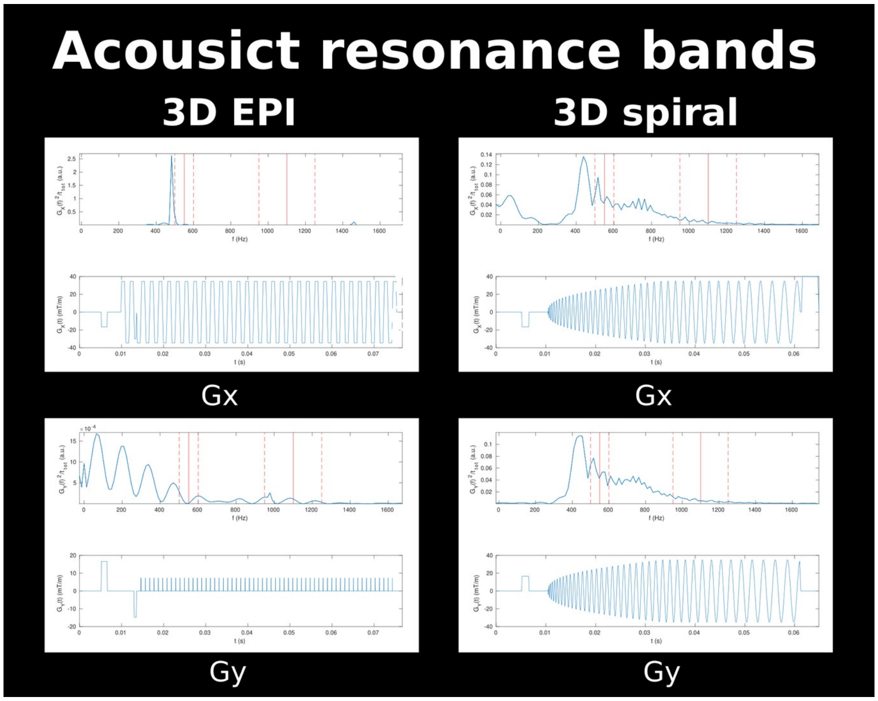

Supplementary figure 2: *Acoustic resonance band analysis for 3D EPI and 3D spiral readouts. For EPI readouts only a single frequency peak is produced, which should not lie in the acoustic resonance frequency bands. For spiral imaging, the frequency spectrum covers wider frequency range, this means that there is always some energy in the acoustic resonance bands. When designing the spiral (choosing G_{max} and Slew Rate), care must be taken to make sure that the minimum amount of energy lies inside the forbidden bands. In this work, we made sure the highest peak in the spiral frequency spectrum was not in any of the acoustic resonance bands.*

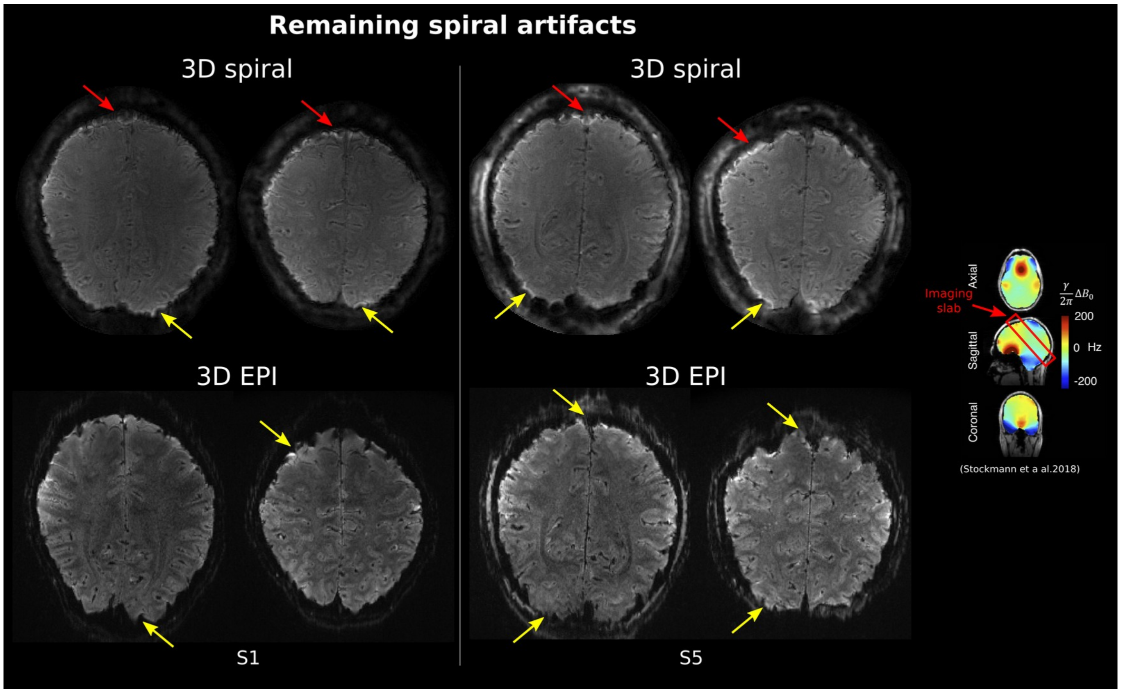

Supplementary figure 3: *Remaining B_0 artifacts in spiral images after full correction. Red arrows point at areas where good shimming is difficult to achieve and high off-resonance values are expected (see figure from Stockmann et al. 2018). Yellow arrows show ring artifacts due to through-plane dephasing or incomplete B_0 mapping and off-resonance correction.*

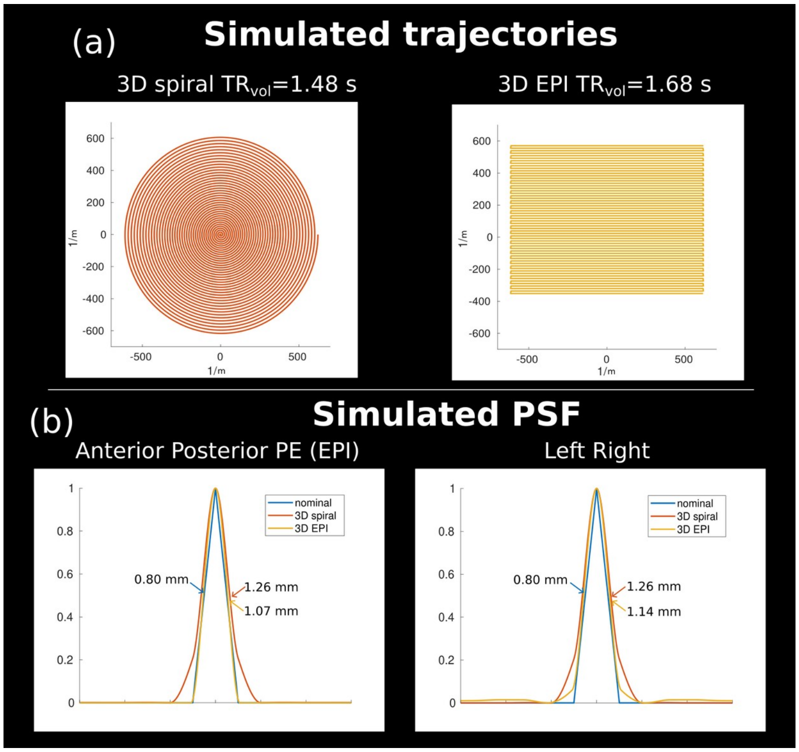

Supplementary figure 4: *Point Spread Function simulations. (a) 3D spiral and 3D EPI trajectories used in this work used for PSF simulations. (b) PSF simulations of the different trajectories, we expect a ~6% reduction on effective resolution on the 3D spiral compared to the 3D EPI one when using T_2^*=25 ms.*
